# Local research productivity, trends, and collaboration among Filipino nuclear medicine physicians: A bibliometric analysis of the Philippine Journal of Nuclear Medicine

**DOI:** 10.22038/aojnmb.2026.93010.1689

**Published:** 2026

**Authors:** John Kenneth V. Gacula, Raniv D. Rojo

**Affiliations:** Division of Nuclear Medicine, Department of Medicine, Philippine General Hospital, University of the Philippines, Manila, Philippines

**Keywords:** Bibliometric analysis, Filipino, Research, Productivity

## Abstract

**Objective(s):**

The Philippine Journal of Nuclear Medicine, the official publication and peer-reviewed journal of the Philippine Society of Nuclear Medicine, serves as a primary repository of research articles by Filipino nuclear medicine physicians. Despite rapid global developments in nuclear medicine, local research output remains underexplored. With 20 volumes and multiple articles published, the PJNM best reflects the situation of nuclear medicine research in the country. This study analyzed and described local research productivity through a bibliometric analysis of PJNM from 2002 to 2025.

**Methods:**

Full-text research articles were retrieved from both physical and online sources. Eligible articles were classified by study type. Bibliographic data such as authorship and institutional affiliation were extracted. Descriptive statistics and bibliometric mapping using VOSviewer were employed to evaluate publication trends, collaboration networks, and keyword occurrences.

**Results:**

A total of 134 full text articles were included. Publication output peaked during 2010-2012, followed by stable but modest productivity in subsequent years. Observational studies (53%) comprised the majority of publications, followed by case report/series (40%), and meta-analyses/systematic review (7%). No experimental studies were published. JM Obaldo was the most prolific author (n=24), while GFL Goco demonstrated the strongest collaborative links (37). St. Luke’s Medical Center-Quezon City produced the highest research output and linkages. Keyword mapping revealed seven thematic clusters, namely: bone imaging, cardiac and parathyroid scintigraphy, radioactive iodine therapy for thyroid cancer, pediatric scintigraphy, prostate cancer and theranostics, MPI SPECT image quality, and renal scintigraphy. The more recent articles focused on oncology, PET/CT, and theranostics.

**Conclusion:**

PJNM reflects the growth and evolving focus of nuclear medicine research in the Philippines. While research output is diverse and increasingly aligned with international trends, there is a need to strengthen experimental and translational studies, foster broader collaborations, and achieve international indexing to enhance visibility and global impact.

## Introduction

 The Philippine Journal of Nuclear Medicine (PJNM; ISSN 1655-9266) is the official publication and peer-reviewed journal of the Philippine Society of Nuclear Medicine (PSNM). Created in 2002, it began circulation in 2003 through the efforts of Dr. Jerry Obaldo, its founding editor-in-chief (EIC) (1). The journal accepts original researches in clinical nuclear medicine and allied fields such as physics, dosimetry, radiation biology, computer science, radio-pharmacy, radiochemistry, and others written by nuclear medicine physicians and allied professionals. In 2010, it was indexed in the Western Pacific Region Index Medicus (WPRIM) (2, 3). Some of the articles and issues are stored and accessible online via the Health Research and Development Information Network (HERDIN), the Philippine national health research repository. As of 2025, 23 years after its maiden issue, PJNM has published 20 volumes containing multiple researches, predominantly by Filipino nuclear medicine physicians. Thus, the journal serves as a key resource to assess local research productivity, topic trends, and collaborations nuclear medicine specialists.

 For academic clinicians, publication metrics are vital indicators of scholarly productivity. Publication sources mostly involve peer-reviewed journals in a single or diverse field of study. These are important in building one’s productivity and are usually used for promotions in the academe, grant applications, or department or university performances (4). 

 Publications are also used in evaluating institutions and countries, as there is a positive correlation between research productivity and health outcomes and organizational performances (5, 6). In bibliometrics, productivity is measured through publication counts. Citation count is also used as a proxy output value for research activity as it shows how the produced knowledge is being disseminated and is used as measure of impact in bibliometrics (7).

 Bibliometric analysis is a popular type of study that aims to provide an overview about a certain topic or field, identify knowledge gaps, and surface novel ideas that may guide researchers to potential topics (8). It involves two factors, namely performance analysis, which is a description of the research constituents (e.g. institutions, authors, countries, etc.) and their contribution to the field; and science mapping, which determines the relationships between constituents (8). These relationships are complex, multidimensional, and tend to be overwhelming with data. The visualization of information facilitates transfer of knowledge through graphic representation (9). With the rising popularity of bibliometric studies, VOSviewer (www.vosviewer.com) was created and has allowed authors to construct bibliometric maps using authors or journals (co-citation data) or keywords (co-occurrence data) for free. It creates “distance-based maps,” from its name, that uses distance to show strength of relation between items through the VOS (visualization of similarities) mapping technique (10).

 Bibliometric analysis could be applied in assessing local research productivity using national research journals. Some have even used this to assess the research productivity of a certain region or country, specifically but not limited to their local journals given its accessibility. Vinardell and Macias (2007) conducted a bibliometric study of the Revista Española de Medicina Nuclear (REMN; Online ISSN 2253-8089), the official journal of the Spanish Society of Nuclear Medicine and Molecular Imaging (www.elsevier.es/es-revista-revista-espanola-medicina-nuclear-e-125). They have identified that there was an increasing trend of “scientific output” in the journal’s 25 years of publication, with notable increase in original studies and submissions from Latin America, brought upon when REMN was included in the Index Medicus/Medline database in 1997 (11). Vijayakumar, et al (2019) conducted a bibliometric analysis of the Indian Journal of Nuclear Medicine (IJMN; Print ISSN: 0972-3919; Online ISSN 0974-0244), the official publication of the Society of Nuclear Medicine, India (https://journals.lww.com/ IJNM). From 2014 to 2018, there was an increasing trend in the number of published papers, notably in 2017 with 15.2% average growth rate (12). The aforementioned studies involving nuclear medicine journals reveal how helpful bibliometric analysis is not only in describing the characteristics and impact of the journal, but also in identifying the trend in research productivity and topics in nuclear medicine in their respective countries.

 Despite the rapid developments worldwide in the field of nuclear medicine, there is little documentation of the state of research productivity in the Philippines. With numerous research articles covering a wide variety of topics since its inception, the PJNM is focal source that may provide a good overview of the current situation of nuclear medicine research in the Philippines. Hence, this study aims to analyze and describe the research productivity in the field of nuclear medicine locally through a bibliometric analysis of the PJNM from 2002 to 2025. Specifically, it aims to: 1) analyze publication trends and article types, 2) identify prolific authors and institutions, 3) map collaboration networks, and 4) assess topic evolution through keyword analysis.

## Methods

### Research Study Design and Data Collection

 Records of the published studies in PJNM were reviewed using available physical and online (2021 to 2025, through the official website of PSNM [www.psnm.ph/journal]) copies. 

 Likewise, the WPRIM of the World Health Organization (WHO) was utilized as reference of the articles published per issue. When unavailable, HERDIN was used to obtain electronic copies.

 Completed studies with full text (either physical or electronic) that were published in the PJNM were included in the analysis. Securing full-text copies allowed us to adequately scrutinize, appropriately categorize, and obtain the complete bibliographic data of each study. 

 Complete names of all authors were recorded, predominantly focused on physicians regardless of their status (trainee, diplomate, fellow, or not) in PSNM at the time of publication. Abstract only articles were excluded along with interesting photos, conference proceedings, letters to the editor or correspondence, editorials, commentaries, and guidelines.

 To ensure that the studies included in the analysis reflected the local research situation, submissions by foreign authors and Filipino authors with international affiliations were excluded, although this may underrepresent total contributions. Identified duplicates were removed prior to analysis.

 Assessment of the records and articles was done independently by each reviewer. Any disagreement was resolved through discussion, and if necessary, a third party was involved to reach a consensus. 

 Articles that fulfilled the set criteria were further categorized into the type of research, namely: case report or series, meta-analysis and/or systematic review, observational study, or experimental. The following details from each article were collected and tabulated:

a. Year and month of publication

b. Volume and issue of PJNM where the article is indent to keep the enumeration

c. Title

d. Authors and their affiliations

e. Type of article 

f. Keywords

 Bibliographic information was extracted and encoded in Microsoft Excel for archiving and descriptive analysis. To create a Research Information Systems (.ris) file, the bibliographic data of each article was manually inputted in Mendeley Reference Manager (version 2.132.1; Elsevier Ltd). Standardization and unification process were applied for the names of the authors and their affiliations for ease of collation and to avoid redundancy. Hyphenated names, if recognized and used, of the married female authors were incorporated.

 Data syntheses and quantitative analysis were done in Excel (Microsoft, USA) using its basic statistical functions including frequency counts of the research articles published and their categories, authors (as primary and co-authors), and affiliated institutions. Visualization was done through graphs and distance maps. As for determination of linkages among authors and keywords, as well as generation of network and overlay visualization, VOSviewer (version 1.6.20) was utilized.

## Results

### Article Retrieval and Selection

**Figure 1:**
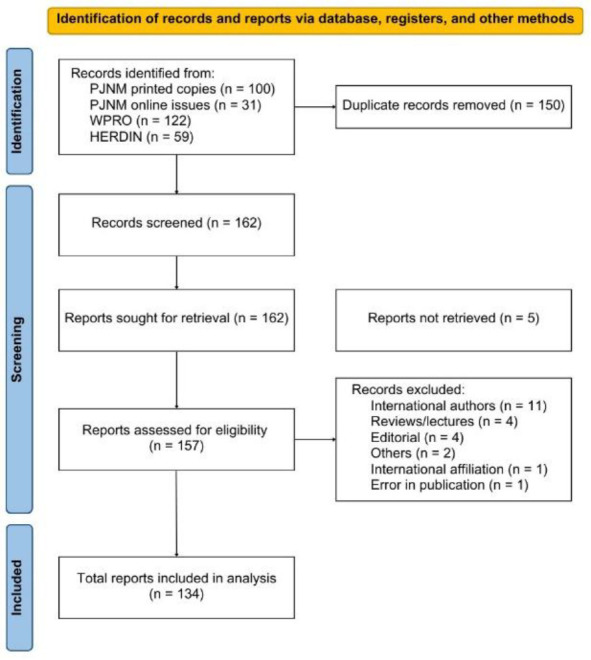
PRISMA flow diagram for the selection of records and reports for analysis from database, registers, and other methods

 A total of 212 records were identified from online databases (WPRO, HERDIN, and PJNM online issues), and 100 records were obtained through manual review of the printed PJNM issues. After removing 150 duplicate records, 162 unique records were assessed for eligibility. Of these, five full texts were unavailable, 22 failed to meet inclusion criteria, and one was excluded as a publication error. In total, 134 full-text articles satisfied all criteria and were included in the final analysis. Figure 1 shows the flow diagram for the identification, screening, and inclusion of articles for analysis.

### Trends in Publications

**Figure 2:**
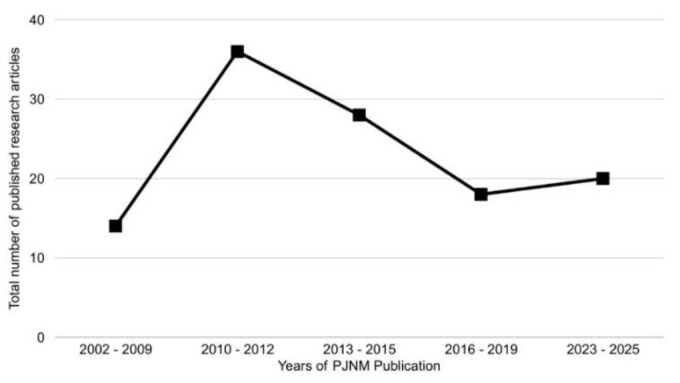
Trend of total published research articles in PJNM per cluster years from 2002 to 2025

 The temporal distribution of publications revealed fluctuating patterns across PJNM’s 23-year history. Figure 2 shows the trend of the published research articles in the PJNM from 2002 to 2025 (clustered every three years, except for 2002 to 2009 and 2016 to 2019). The journal’s early years (2002, 2008-2009) were characterized by low productivity, with only 14 articles published in its first three years. A marked increase occurred between 2010 and 2012, when the number of articles peaked at 36, suggesting heightened research activity during this period. Subsequent years showed a gradual decline, but a relatively stable output compared to the initial decade. Overall output has demonstrated resilience, particularly in recent years where annual numbers have been maintained at modest yet steady levels

.

### Types of Research Articles

**Figure 3:**
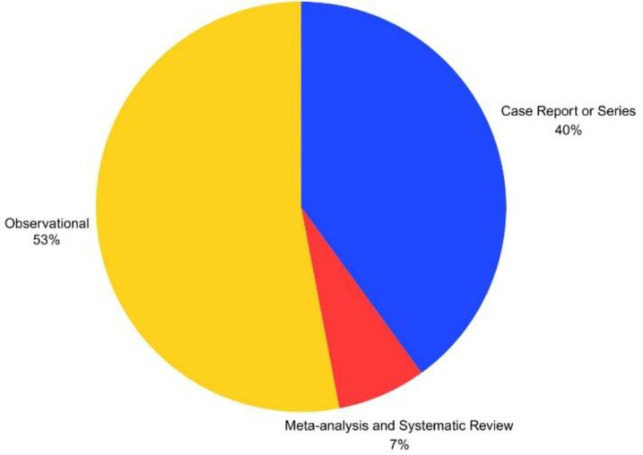
Distribution of each research category based on the total number of research articles published in PJNM (2002-2025)

 The included articles were categorized into their research type: case report or series, meta-analysis and systematic review, observational, and experimental studies. Figure 3 shows the distribution of each category to the total number of articles included in the analysis (n=134). Observational studies dominated the journal’s output, accounting for 53% (n=71) of all publications. Case reports and case series

 followed at 40% (n=53), demonstrating the continued importance of clinical documentation in local research. Meta-analyses and systematic reviews represented only 7% (n=10), reflecting a limited focus on evidence synthesis. Notably, no experimental studies were identified across the entire publication period, underscoring the scarcity of interventional or laboratory-based research within PJNM.

**Figure 4:**
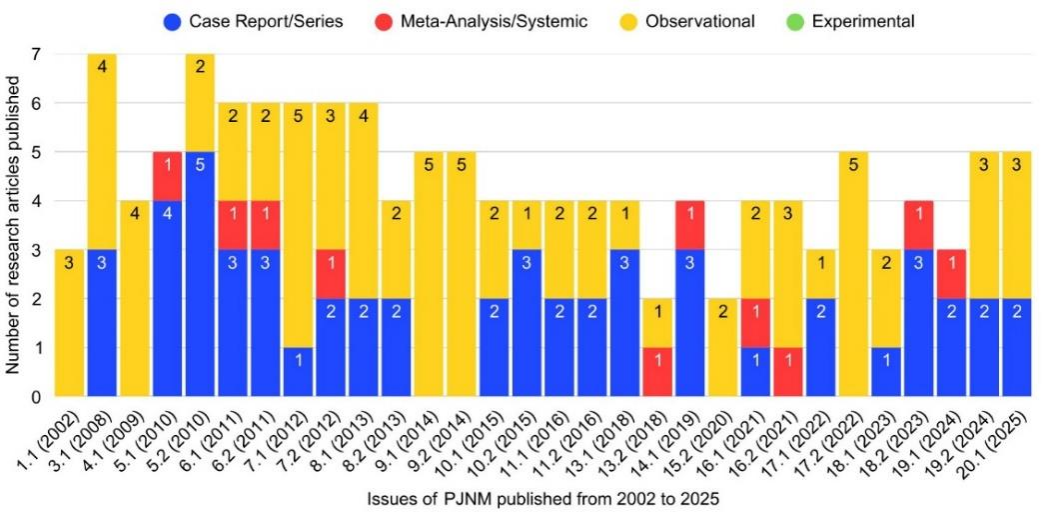
Distribution of research categories per each issue of PJNM published from 2002 to 2025

Figure 4 shows the trend and distribution of each category per issue. PJNM volume 3 issue 1 and volume 5 and issue 2 had the greatest number of research articles published (n=7). Notably, temporal patterns also varied by article type. Earlier years tended to publish more case reports, while later years demonstrated an increase in observational studies and reviews, suggesting gradual maturation of research methodologies within the local nuclear medicine community.

### Most Prolific Authors

 Authorship analysis revealed key contributors to the journal’s output. Table 1 summarizes the names of the five individuals with the most first author papers. As first authors, PA Bautista-Peñalosa and PEA Fernando led with five publications each (3.7%). CU Liao, JC Mendoza, and DD Tumapon each contributed four articles (3.0%). When all authorships were considered, JM Obaldo emerged as the most prolific contributor, credited in 24 articles (17.9% of all publications). Other highly productive authors included GFL Goco (15 articles, 11.2%), IS Bandong (13, 9.7%), PA Bautista-Peñalosa (13, 9.7%), and VPC Magboo (13, 9.7%). Table 2 presents the five persons that authored the greatest number of articles.

**Table 1:** Top 5 most prolific authors by first authorship among the analyzed research articles

**Rank**	**Author**	**Documents**	**Percentage**
1.5	Bautista-Peñalosa, PA	5	3.7%
1.5	Fernando, PEA	5	3.7%
4	Liao, CU	4	3.0%
4	Mendoza, JC	4	3.0%
4	Tumapon, DD	4	3.0%

**Table 2:** Top 5 most prolific authors among the analyzed research articles

**Rank**	**Author**	**Documents**	**Percentage**
1	Obaldo, JM	24	17.9%
2	Goco, GFL	15	11.2%
4	Bandong, IS	13	9.7%
4	Bautista-Peñalosa, PA	13	9.7%
4	Magboo, VPC	13	9.7%

### Collaboration Networks

**Figure 5:**
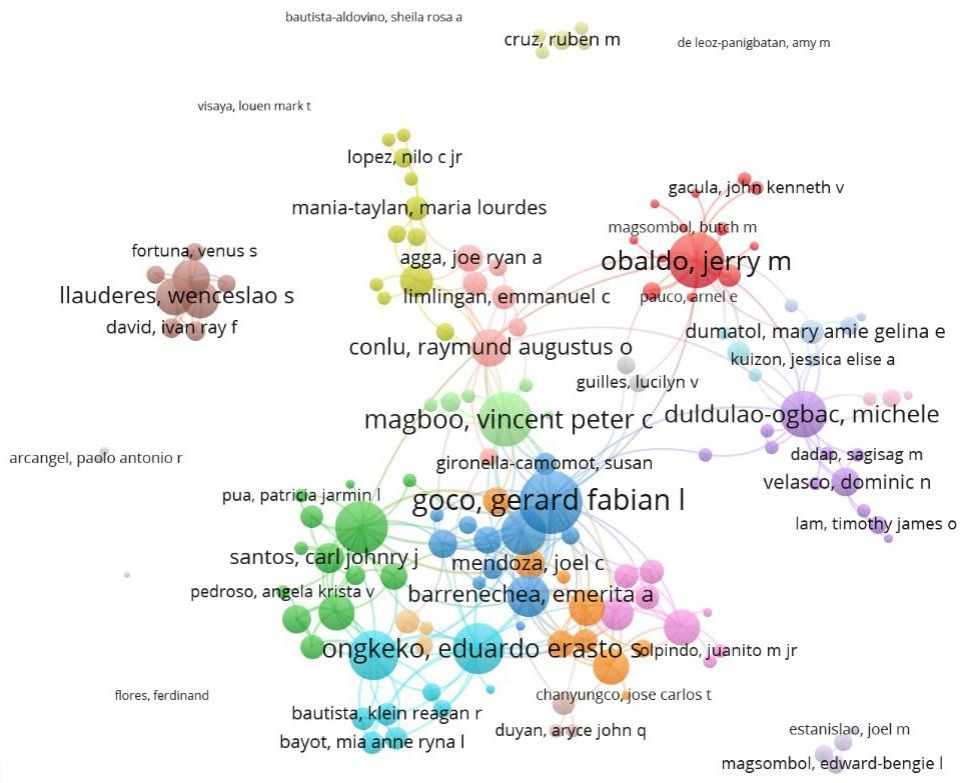
Visualization of the linkages, measured by total link strength, among the authors of the published research articles in PJNM; size of the circle is proportional to the number of articles containing the indicated keyword in the network

 Co-authorship mapping revealed varying degrees of collaboration among nuclear medicine physicians. Table 3 summarizes the five authors with the strongest collaborations, using total link strength. GFL Goco demonstrated the strongest collaborative network, with a total link strength of 37 across three clusters of co-authors. JM Obaldo (30), VPC Magboo (27), IS Bandong (26), and EES Ongkeko (25) also showed substantial collaborative strength. These patterns suggest that while individual productivity is important, influence within the field is equally shaped by breadth of collaborative engagement. Figure 5 illustrates how certain authors function as central nodes, linking multiple contributors across institutional boundaries.

**Table 3:** Top 5 authors with the strongest link among other co-authors

**Rank**	**Author**	**Total Link Strength**	**Cluster**
1	Goco, GFL	37	3
2	Obaldo, JM	30	1
3	Magboo, VPC	27	11
4	Bandong, IS	26	2
5	Ongkeko, EES	25	6

### Institutional Productivity and Linkages

Table 4 summarizes the affiliations with greatest number of research articles produced and published. Institutional analysis demon-strated that nuclear medicine residency training centers are the most productive sources of research. Authors affiliated with the Department of Nuclear Medicine at St. Luke’s Medical Center–Quezon City (SLMC-QC) led by a wide margin, contributing 49 articles (36.6%). The Philippine Heart Center (PHC) followed with 28 publications (20.9%), and Cardinal Santos Medical Center with 13 publications (9.7%), while St. Luke’s Medical Center–Global City (SLMC-GC) and the University of Santo Tomas Hospital (USTH) each produced 12 publications (9.0%). Academic institutions had limited but notable output, including the University of the Philippines Manila (UPM; n=5, 3.7%) and De La Salle University Manila (DLSU; n=1, 0.7%). Most of the institutions are located in the National Capital Region (NCR), with only Bethany Hospital in La Union and Careview PET/CT Center – Mary Mediatrix Medical Center in Batangas as the affiliations located in Region I and IV, respectively. Collectively, these results underscore the dominant role of training hospitals in Philippine nuclear medicine research, with academic–clinical partnerships contributing more modestly.

**Figure 6:**
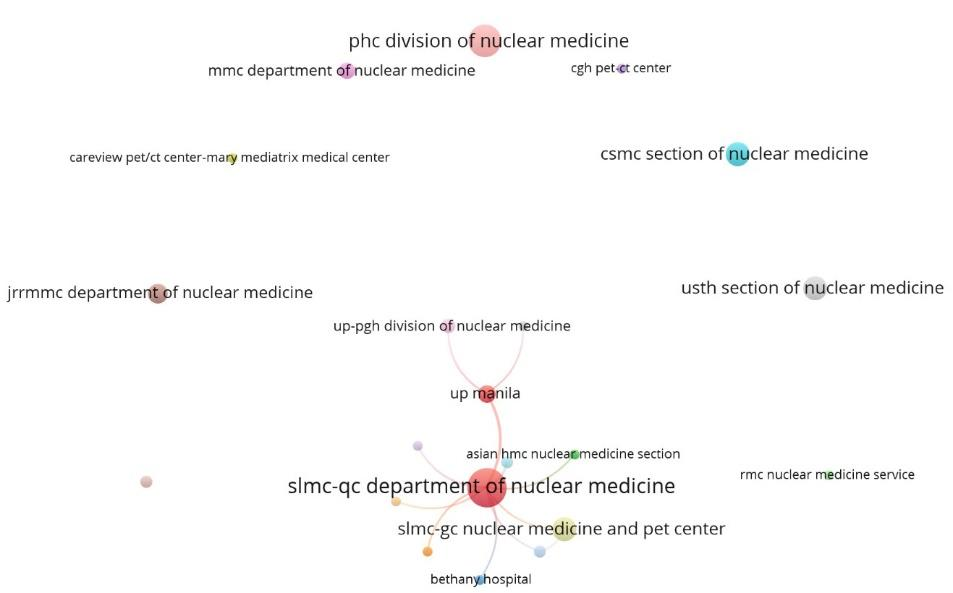
Visualization of the affiliations and their linkages based from the research articles published in PJNM; size of the circle is proportional to the number of articles containing the indicated keyword in the network

Figure 6 visualizes the affiliations and their linkages (based on occurrence) with others based on the research articles analyzed. The Department of Nuclear Medicine of SLMC-QC had the greatest number and strongest linkages with other institutions, highlighting SLMC-QC as a central hub for institutional research.

**Table 4:** Affiliations with greatest number of research articles produced and published in the PJNM

**Rank**	**Affiliation**	**Region**	**Type**	**Rec**	**%**
1	Department of Nuclear Medicine, St Luke’s Medical Center – Quezon City	NCR	Hospital	49	36.6%
2	Division of Nuclear Medicine, Philippine Heart Center	NCR	Hospital	28	20.9%
3	Department of Nuclear Medicine, Cardinal Santos Medical Center	NCR	Hospital	13	9.7%
4.5	Department of Nuclear Medicine and PET/CT Center, St Luke’s Medical Center – Global	NCR	Hospital	12	9.0%
4.5	Section of Nuclear Medicine, University of Santo Tomas Hospital	NCR	Hospital	12	9.0%
6	Division of Nuclear Medicine, Jose R Reyes Memorial Medical Center	NCR	Hospital	7	5.2%
7	University of the Philippines Manila	NCR	Academic	5	3.7%
8	Department of Nuclear Medicine, Makati Medical Center	NCR	Hospital	4	3.0%
9	Division of Nuclear Medicine, UP-Philippine General Hospital	NCR	Hospital	3	2.2%
11	Institute of Radiology, St Luke’s Medical Center – Global City	NCR	Hospital	2	1.5%
11	Institute of Radiology, St Luke’s Medical Center – Quezon City	NCR	Hospital	2	1.5%
11	Section of Nuclear Cardiology, Heart Institute, St Luke’s Medical Center – Quezon City	NCR	Hospital	2	1.5%
17	Bethany Hospital, San Fernando, La Union	I	Hospital	1	0.7%
17	Careview PET/CT Center – Mary Mediatrix Medical Center	IV	Hospital	1	0.7%
17	De La Salle University – Manila	NCR	Academic	1	0.7%
17	Institute of Ophthalmology, St Luke’s Medical Center	NCR	Hospital	1	0.7%
17	Institute of Surgery, St Luke’s Medical Center	NCR	Hospital	1	0.7%
17	Nuclear Medicine Section, Asian Hospital and Medical Center	NCR	Hospital	1	0.7%
17	Nuclear Medicine Service, Rizal Medical Center	NCR	Hospital	1	0.7%
17	PET/CT Center, Cancer Institute, Chinese General Hospital and Medical Center	NCR	Hospital	1	0.7%
17	Section of Nuclear Medicine, University of Perpetual Help Medical Center	NCR	Hospital	1	0.7%

### Keyword Analysis and Thematic Clusters

**Figure 7:**
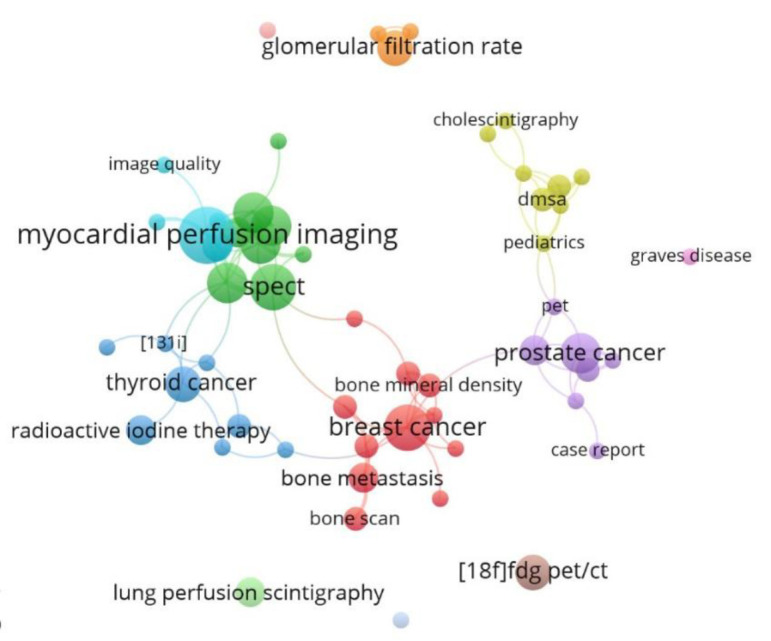
Visualization of the keywords used in the research articles published in PJNM; size of the circle is proportional to the number of articles containing the indicated keyword in the network

**Figure 8:**
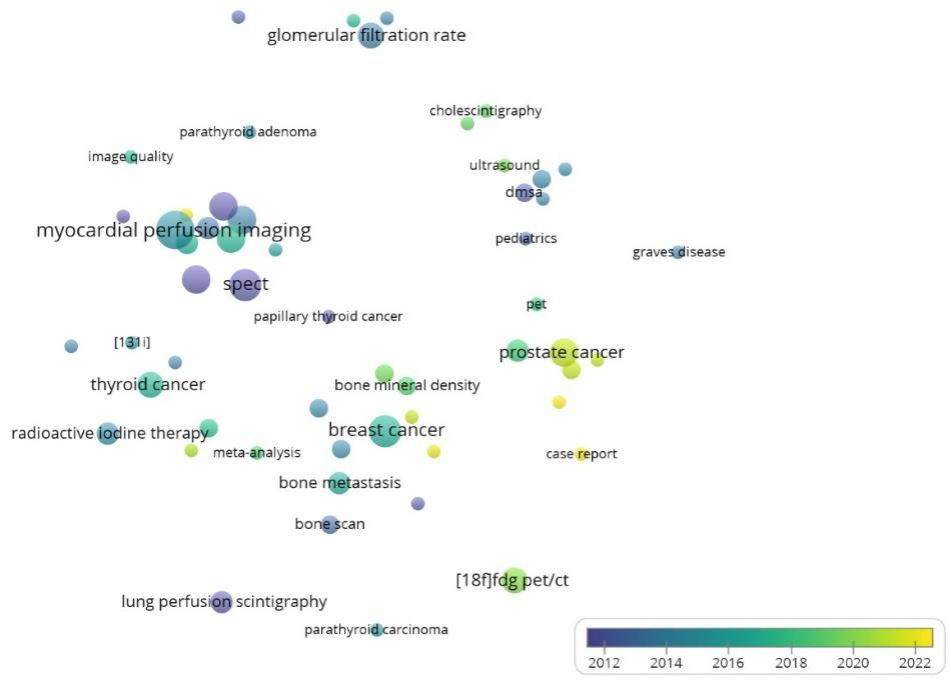
Visualization of the occurrences of the keywords based on the year of their publication; dark blue shade represents occurrences in the older years, while the lighter shades of green and yellow signifies more recent publication

Figure 7 shows the keywords used in the articles included in the analysis, their strength based on occurrence, and connections with other keywords. Table 5 shows the clusters formed after analysis of these keywords. A total of seven keyword clusters emerged from VOSviewer analysis, reflecting the thematic diversity of PJNM articles. Cluster 1 (red) focused on bone imaging, osteoporosis, and cancer metastases; Cluster 2 (green) on myocardial perfusion imaging (MPI) and parathyroid scintigraphy; Cluster 3 (blue) on radioactive iodine (RAI) therapy for thyroid cancer; Cluster 4 (yellow) on pediatric scintigraphy such as renal cortical scans and vesicoureteral reflux; Cluster 5 (purple) on prostate cancer and theranostics (e.g., prostate-specific membrane antigen [PSMA] positron emission tomography/computed tomography [PET/CT] and [^177^Lu] Lu-PSMA therapy); Cluster 6 (cyan) on myocardial perfusion single-photon emission computed tomography (SPECT) image quality; and Cluster 7 (orange) on renal function measurement with diethylenetriamine pentaacetate (DTPA) scintigraphy. Temporal overlay visualization (Figure 8) showed that early years (2002–2009) were dominated by bone and cardiac scintigraphy, while later years (2016–2025) increasingly focused on oncologic imaging, PET/CT, and theranostics.

**Table 5:** Seven biggest clusters formed, their respective colors, and included keywords

**Cluster & Color**	**Keywords or Topics**
Cluster 1 (Red)	[^18^F] FDG PET [^99m^Tc] Tc-HDP Bone metastasis Bone mineral density Bone scan Bone scintigraphy Breast cancer DXA Filipino Osteoporosis Papillary thyroid cancer
Cluster 2 (Green)	[^201^Tl] [^99m^Tc] Tc-sestamibi Coronary artery disease Coronary flow reserve Dipyridamole Myocardial perfusion scan Parathyroid adenoma SPECT
Cluster 3 (Blue)	[^131^I] Meta-analysis Radioactive iodine Radioactive iodine therapy RAI therapy Thyroid cancer Well-differentiated thyroid cancer Whole body scan
Cluster 4 (Yellow)	Cholescintigraphy DMSA HIDA Pediatrics Renal cortical scan Ultrasound Urinary tract infection Vesicoureteral reflux
Cluster 5 (Purple)	[^177^Lu] Lu-PSMA-617 [^68^Ga] Ga-PSMA-11 Case report PET PET/CT Positron emission tomography Prostate cancer
Cluster 6 (Cyan)	[^99m^Tc] Tc-Tetrofosmin Cedars-Sinai Quantitative Gated SPECT Image quality Myocardial perfusion imaging SPECT/CT
Cluster 7 (Orange)	[^99m^Tc] Tc-DTPA Glomerular filtration rate Inoue method

## Discussion

 Our study aims to analyze and describe the research productivity, trends, and collaboration among Filipino nuclear medicine physicians through a bibliometric analysis of the research articles published in the PJNM from 2002 to 2025. Through this study, we have identified the number, trend, and categories of the research articles published in the journal since its first issue. Furthermore, we have identified the most prolific authors, contributors, and affiliations that have produced and published these research articles. The prevalence and trend of topics were also identified through the use of occurrence and connections of keywords utilized.

 The PJNM was imagined to be the response to the perceived lack of a dedicated journal to publish studies by local nuclear medicine trainees and practitioners (1). As JM Obaldo (2002) stated in his first editorial, the “sheer absence” of publications undermines the credibility of scientific and academic circles, much like of PSNM and nuclear medicine in the Philippines at that time. Other than addressing this problem, the creation of the journal by PSNM was also seen as an encouragement for local nuclear medicine research to flourish (13).

 It was evident that the journal struggled in its initial years (2002 to 2009) as it recorded the lowest number of published research articles. It was in 2008, with GFL Goco as EIC, that the journal published two issues that year, signifying the increase in interest, research activities, and submissions (14). This momentum led to the peak of the journal in 2010 to 2012. This was followed by a slow decline afterwards, but still relatively better than its maiden years. The sustained number of submitted and published research articles could be related with the increasing number of nuclear medicine training programs, trainees, and certified specialists in the country as more researches are being produced yearly. This trajectory parallels international experiences: in Spain and India, journal productivity also improved after indexing milestones (11, 12). 

 However, PJNM remains limited by lack of inclusion in Scopus or PubMed, underscoring the importance of pursuing indexing for growth.

 The PSNM and the institutions with accredited nuclear medicine residency program require trainees to complete and present two research projects as part of their training and promotion, and to demonstrate competence in practice-based learning and communication skills (15). 

 Certified nuclear medicine specialists, classified as “diplomates” by the PSNM, could elevate their status to “fellow” by fulfilling certain requirements, involving at least one published scientific paper (3).

 Observational studies were the most published in PJNM, accounting for 53% of the articles published. This was followed by case report or series (40%) and meta-analysis (7%). No true experimental study has been published in the journal. This dominance of descriptive work highlights the need to support more experimental and translational studies to align with global nuclear medicine research (16, 17). 

 We have identified the most prolific nuclear medicine physicians that have written researches as first author. Four (PA Bautista- Peñalosa, PEA Fernando, CU Liao, and JC Mendoza) of which have achieved “fellow” status in PSNM and majority of their works have been affiliated with their home training institution or where they serve as consultant mentor. This trend is further observed as we identified the authors with greatest number of articles authored and collaboration with other nuclear medicine physicians. JM Obaldo, with 24 papers authored, has been a mentor and co-author with trainees in two training hospitals, University of the Philippines-Philippine General Hospital (UP-PGH) and Philippine Heart Center (PHC). However, it was GFL Goco, affiliated with SLMC-QC that recorded the strongest and greatest number of linkages and collaborations with 15 research articles. This distinction illustrates that productivity (article count) and collaboration (link strength) are different but complementary markers of influence (18, 19). Broad co-authorship networks, as shown in other bibliometric analyses, often translate to greater long-term visibility and impact.

 In bibliometric mapping, it is crucial to understand that the number of authored papers does not directly correlate with an author's link strength within a research network. Co-authorship networks reflect the collaborative relationships among researchers, indicating that link strength calculations are based on the number of connections one maintains rather than merely publication counts (20). 

 VOSviewer, as demonstrated by Ageel, facilitates the visualization of these bibliometric networks, helping researchers identify collaboration patterns through co-authorship interactions (21). The distinction between full counting and fractional counting in bibliometric analysis allows for nuanced measurements of collaborative engagement that consider the cumulative weight of co-authorship ties (18). 

 Therefore, while an author may have a higher output of papers, their limited collaboration scope can result in less link strength compared to another researcher with fewer publications but broader co-authorship ties, as supported by the evidence of bibliometric dynamics presented in the literature (19).

 Among the affiliations with greatest number of research articles produced and published, we have identified that majority of them are hospitals with nuclear medicine residency training programs. Training institutions remain crucial engines of research across disciplines; a phenomenon clearly reflected in the field of nuclear medicine in the Philippines through centers such as the PHC and SLMC. In fact, research engagement and output are formal requirements in Philippine nuclear medicine residency programs, where scholarly activity is actively encouraged as part of training. This emphasis is aligned with the objectives outlined in the guiding principles for theranostics education and practice (22). The literature consistently demonstrates that residency programs not only foster clinical expertise but also enhance research productivity, largely through structured mentorship, mandated research objectives, and robust collaborative networks (23, 24). Similar patterns are evident internationally, where residency and fellowship programs in the health sciences substantially influence both the volume and quality of research output (25, 26). For example, Layon et al. (2017) highlighted that formal research training during residency strengthens academic contributions, with dedicated research components leading to increased scholarly activity (27). Likewise, other studies underscore how structured environments nurture early exposure to research methodology, positioning residents to engage more actively in academic networks and to generate work that meaningfully informs policy and practice (26, 28). Collectively, these finding underscore that educational institutions are not merely passive avenues for training but are pivotal contributors to the advancement of the research ecosystem.

 Other than producing and publishing the greatest number of research articles in PJNM, SLMC-QC also had the greatest number of linkages: nine with other affiliations and a total link strength of 11. Its greatest ties are linked with the nuclear medicine department of its sister hospital, St. Luke’s Medical Center Global City (SLMC-GC), possibly due to the doctors that also serve as mentors in both institutions, leading to knowledge expansion and transfer between the two institutions. Three other collaborations are within the same hospital, namely with the Institute of Surgery, the Institute of Radiology, and the Eye Institute of SLMC-QC. This interdisciplinary approach in research allows breaking down of disciplinary barriers that would allow experts to share research goals and experiments that may lead to potential solutions (29). SLMC-QC also recorded to have linkages with two academic institutions, DLSU and UPM. This diverse partnership with educational institutions emphasizes the need for localization and sustainability of such partnership for opportunities, needs, and resources. The academic institutions could see this as an opportunity to find potential participants, grants and publications, while for healthcare institutions, it could be for development of research skills and research activities to improve patient outcomes (30). 

 This linkage is also present with UP-PGH and UPM, specifically Department of Physical Sciences and Mathematics, which are units within the same university.

 Most research articles were produced and published by institutions based in the National Capital Region, likely because most of the training centers and nuclear medicine physicians are concentrated in this region. NCR also contains the largest number of hospitals equipped with nuclear medicine facilities, leading to better patient access and research opportunities. A similar pattern has been observed in the research productivity of Filipino neurologists as most of their research outputs are concentrated in NCR and adjacent Region IV-A, both being considered most populous and more developed regions in the country (31).

 Keyword visualization identified seven major thematic clusters among PJNM publications. Cluster 1 (red) the largest group, reflects the foundational role of bone imaging in nuclear medicine practice. Dual-energy X-ray absorptiometry (DXA) remains a standard in osteoporosis screening, while bone scintigraphy continues to be indispensable in detecting skeletal metastases, particularly in breast and thyroid cancers. This aligns with global literature, where bone imaging has historically dominated nuclear medicine output due to its accessibility and broad clinical utility (16, 17).

 Cluster 2 (green) demonstrates the importance of [^99m^Tc] Tc-sestamibi in both parathyroid and cardiac scintigraphy. MPI with [^99m^Tc]-labeled tracers remains one of the most frequently performed nuclear medicine procedures worldwide, reflecting its pivotal role in the diagnosis of coronary artery disease (32, 33).

 Cluster 3 (blue) highlights radioactive iodine (RAI) therapy for differentiated thyroid carcinoma, a cornerstone of nuclear medicine in the Philippines. Given the country’s relatively high incidence of thyroid disease, it is unsurprising that this topic occupies a central position in PJNM (34).

 Cluster 4 (yellow) focuses predominantly on pediatric imaging, including renal cortical scintigraphy, vesicoureteral reflux studies, and hepatobiliary scintigraphy. Pediatric nuclear medicine represents a niche but vital field, providing functional information where anatomic imaging alone may be insufficient. 

 Internationally, pediatric-focused bibliometric analyses have similarly noted the pre-dominance of renal and hepatobiliary scintigraphy in children (16, 32). 

 Cluster 5 (purple) represents one of the most recent and rapidly expanding areas of nuclear medicine: prostate cancer theranostics. The introduction of [^68^Ga] Ga-PSMA-11 PET/CT for staging and [^177^Lu] Lu-PSMA-617 for targeted radionuclide therapy has revolutionized prostate cancer management globally (35). Its appearance as a distinct cluster in PJNM reflects the Philippines’ adoption of cutting-edge molecular imaging, though published output remains limited compared to international literature (32, 33).

 Cluster 6 (cyan), thematically related to Cluster 3, underscores methodological concerns such as image quality in MPI. The attention to [^99m^Tc] Tc-Tetrofosmin reflects both practical issues of radiotracer selection and the ongoing need to optimize image acquisition in local practice.

 Finally, cluster 7 (orange) centers on renal function assessment using [^99m^Tc] Tc-DTPA. This cluster highlights the clinical importance of renal scintigraphy in the Philippine setting, where kidney disease remains highly prevalent (36). Nuclear medicine offers valuable tools for functional assessment that complement conventional laboratory and imaging methods (37).

 Early PJNM publications were dominated by bone, cardiac, pulmonary, and renal scintigraphy, reflecting the core practices of nuclear medicine during that period. In contrast, more recent years have shown a clear shift toward oncologic imaging – particularly thyroid, breast, and prostate cancer – as well as the increasing use of PET/CT and theranostics with tracers such as [^18^F] FDG, [^68^Ga] Ga-PSMA-11, and [^177^Lu] Lu-PSMA-617. This thematic shift indicates that PJNM is gradually aligning with international trends in nuclear medicine research, though the volume of output and the absence of experimental designs suggest that further development and support remain necessary (32, 33).

 The PJNM and local research of nuclear medicine remain evolving through time. Nuclear medicine is strongly tied with oncology, as it is predominantly tackled in the top-cited and top Altmetric articles in the field (33). Given its vital role in oncologic imaging, research about PET is a popular and continuously growing topic in nuclear medicine (17, 33). We have identified that these topics are just emerging in the local nuclear medicine research scene. The Philippines had its first PET/CT scanner in SLMC-QC in 2008, and the first centralized cyclotron facility with PET/CT center in National Kidney and Transplant Institute (NKTI) in 2016 (3). The country is not entirely behind and is trying to catch up with the latest developments in oncology and PET imaging, as well as theranostics, with several medical institutions, both private and government-owned, setting-up their own PET/CT centers. Further encouragement and assistance may be needed to invite more nuclear medicine physicians to produce and publish articles covering not only the aforementioned topics but also the emerging developments in the field.

 This may not be the first bibliometric study of a local journal, but this is the first to analyze the local research productivity and trends in nuclear medicine in the Philippines through a bibliometric analysis of a local journal, the PJNM. The role of local journals in sustaining national research ecosystems is increasingly recognized, especially in developing countries like the Philippines. While international journals often dominate citation indices and academic prestige, local journals serve as indispensable platforms for sharing regionally relevant research. For instance, the PJNM not only publishes essential clinical audits and innovative research methodologies but also strengthens the connection between researchers and healthcare practitioners by disseminating findings pertinent to local medical practices. Thus, indexing PJNM in Scopus or PubMed Central should be a priority to improve international reach (38, 39).

 Journals such as Acta Medica Philippina (ActaMedPhillip, ISSN 0001-6071) and the Philippine Journal of Internal Medicine (PJIM, ISSN 0119-9641) exemplify the multifaceted contributions of local publications, offering platforms for diverse research. They capture studies across various disciplines, focusing on issues that may not align with the broader, global scopes of international journals (38). 

 Consequently, local journals not only promote scientific inquiry but also address vital concerns relevant to the Philippine context, such as endemic diseases and healthcare resource allocation. This local focus ensures that unique challenges and conditions specific to the Philippines are documented and addressed in scientific literature. The integration of local journals into national academic ecosystems not only bolsters research output but also aligns with government policies aimed at enhancing the research productivity of higher education institutions (38, 40). For early-career researchers and residents-in-training, local journals provide accessible venues for publication, essential for professional development and academic growth (41). The peer review processes in these journals often place emphasis on local context, ensuring that region-specific variables are accounted for, which is critical for the applicability of research findings to practice (38). Moreover, local journals contribute to the cultivation of a self-sustaining research culture by encouraging young researchers to engage in scholarly activities, ultimately influencing policy developments and clinical practice (39, 42). 

 Further, the importance of local journals extends beyond academics; they are crucial for building capacity in research communities. By fostering an environment where local issues are discussed and documented, these journals help establish a robust framework for knowledge dissemination that is tailored to local needs. This enhances not only the volume of published research but also its relevance and impact on health outcomes (38).

 Despite the breadth of data analyzed, this bibliometric analysis has several limitations. First, only retrieved full-text articles were included, leading to the exclusion of some issues and individual studies. Second, the absence of keywords in certain articles limited the comprehensiveness of the keyword mapping and visualization. Third, because PJNM is not indexed in Scopus or similar databases, citation coupling and impact analysis could not be performed. Finally, the exclusion of Filipino authors with international affiliations may have underestimated the extent of global collaborations. Future research should expand the scope of bibliometric analyses by incorporating articles authored by Filipino nuclear medicine physicians published in international journals, which would provide a more holistic view of both local and global contributions. 

## Conclusion

 This bibliometric analysis of the Philippine Journal of Nuclear Medicine provides the first comprehensive overview of research productivity, trends, and collaborations in Philippine nuclear medicine. The journal and local nuclear medicine research peaked in 2010-2012 and currently exhibits sustained trend, reflecting steady but modest productivity. Most publications were observational in design, with an absence of experimental or translational studies, underscoring the need to diversify research methodologies. Authorship and institutional mapping highlighted the central role of residency training programs and key institutions in driving scholarly output and collaboration. There are diverse themes that are predominant in PJNM with thematic analysis revealing an evolution from traditional bone and cardiac imaging toward oncologic applications, PET/CT, and theranostics, demonstrating increasing alignment with global nuclear medicine research. To sustain this trajectory, Philippine nuclear medicine must strengthen multi-institutional collaborations, expand beyond observational research into experimental and translational studies, and prioritize international indexing of PJNM to enhance visibility. By addressing these areas, PJNM can further solidify its role as the primary platform for advancing nuclear medicine research in the country while positioning Filipino contributions within the global landscape. This study provided a perspective to the current scenario of Philippine nuclear medicine research, and where it should be heading in the next few years.
